# Sex-related differences among patients undergoing surgical aortic valve replacement—a propensity score matched study

**DOI:** 10.1093/icvts/ivae140

**Published:** 2024-08-10

**Authors:** Andreas Zierer, Ruggero De Paulis, Farhad Bakhtiary, Ali El-Sayed Ahmad, Martin Andreas, Rüdiger Autschbach, Peter Benedikt, Konrad Binder, Nikolaos Bonaros, Michael Borger, Thierry Bourguignon, Sergio Canovas, Enrico Coscioni, Francois Dagenais, Philippe Demers, Oliver Dewald, Richard Feyrer, Hans-Joachim Geißler, Martin Grabenwöger, Jürg Grünenfelder, Sami Kueri, Ka Yan Lam, Thierry Langanay, Günther Laufer, Wouter Van Leeuwen, Rainer Leyh, Andreas Liebold, Giovanni Mariscalco, Parwis Massoudy, Arash Mehdiani, Renzo Pessotto, Francesco Pollari, Gianluca Polvani, Alessandro Ricci, Jean-Christian Roussel, Saad Salamate, Matthias Siepe, Pierluigi Stefano, Justus Strauch, Alexis Theron, Andreas Vötsch, Alberto Weber, Olaf Wendler, Matthias Thielmann, Matthias Eden, Beate Botta, Peter Bramlage, Bart Meuris

**Affiliations:** Department of Cardiac, Vascular and Thoracic Surgery, Kepler University Hospital Linz, Linz, Austria; Department of Cardiac, Vascular and Thoracic Surgery, Hospital Wels-Grieskirchen, Wels, Austria; Department of Cardiac Surgery, European Hospital, Rome, Italy; Department of Cardiac Surgery, University Hospital Bonn, Bonn, Germany; Department of Cardiac Surgery, University Hospital Bonn, Bonn, Germany; Department of Cardiac Surgery, Medical University of Vienna, Vienna, Austria; Department of Thoracic and Cardiovascular Surgery, University Hospital RWTH Aachen, Aachen, Germany; Department of Cardiac, Vascular and Thoracic Surgery, Kepler University Hospital Linz, Linz, Austria; Department of Cardiac, Vascular and Thoracic Surgery, Hospital Wels-Grieskirchen, Wels, Austria; Department of Cardiac Surgery, University Hospital St Poelten, St Poelten, Austria; Department of Cardiac Surgery, Medical University of Innsbruck, Innsbruck, Austria; Department of Cardiac Surgery, Leipzig Heart Center, Leipzig, Germany; Department of Cardiology and Cardiac Surgery, Tours University Hospital, Tours, France; Cardiovascular Surgery Department, Hospital University Virgen de la Arrixaca, Murcia, Spain; Department of Cardiac Surgery, University Hospital San Giovanni di Dio e Ruggi d'Aragona, Salerno, Italy; Department of Cardiac Surgery, Institut Universitaire de Cardiologie et de Pneumologie de Québec, Université Laval, Québec City, Québec, Canada; Department of Surgery, Montreal Heart Institute, University of Montreal, Montreal, Canada; Department of Pediatric Cardiac Surgery, University Hospital Erlangen, Erlangen, Germany; Department of Cardiac Surgery, Clinic for Cardiovascular Surgery, Central Military Hospital, Koblenz, Germany; Department of Cardiac, Vascular and Thoracic Surgery, Kepler University Hospital Linz, Linz, Austria; Department of Cardiac, Vascular and Thoracic Surgery, Hospital Wels-Grieskirchen, Wels, Austria; Department of Cardiovascular Surgery, Clinic Floridsdorf, Vienna, Austria; Department of Cardiac Surgery, Heart Clinic Zurich, Hirslanden Klinik, Zurich, Switzerland; Department of Cardiovascular Surgery, University Heart Center Freiburg Bad Krozingen, Bad Krozingen, Germany; Department of Cardiothoracic Surgery, Catharina Hospital Eindhoven, Eindhoven, Netherlands; Department of Thoracic and Cardiovascular Surgery, Rennes University Hospital Center, Rennes, France; Department of Cardiac Surgery, Medical University of Vienna, Vienna, Austria; Department of Cardiothoracic Surgery, Erasmus MC University Medical Center, Rotterdam, Netherlands; Department of Thoracic and Cardiovascular Surgery, University of Wuerzburg, Wuerzburg, Germany; Department of Cardiac Surgery, University of Ulm Medical Center, Ulm, Germany; Department of Cardiac Surgery, National Institute for Health Research Leicester Biomedical Research Centre, Glenfield Hospital, Leicester, UK; Department of Cardiac Surgery, Klinikum Passau, Passau, Germany; Department of Cardiac Surgery, University Hospital Duesseldorf, Duesseldorf, Germany; Department of Thoracic and Cardiovascular Surgery, West-German Heart and Vascular Center, University Duisburg-Essen, Essen, Germany; Department of Cardiothoracic Surgery, Royal Infirmary of Edinburgh, Edinburgh, UK; Department of Cardiac Surgery, Klinikum Nürnberg-Paracelsus Medical University, Nuremberg, Germany; Department of Cardiovascular Surgery, Centro Cardiologico Monzino IRCCS, Milan, Italy; Department of Cardiac Surgery, European Hospital, Rome, Italy; Department of Thoracic and cardiovascular surgery, CHU Nantes, Nantes, France; Department of Cardiac Surgery, University Hospital Bonn, Bonn, Germany; Department of Cardiovascular Surgery, University Heart Center Freiburg Bad Krozingen, Bad Krozingen, Germany; Department of Cardiac Surgery, University Hospital Bern, University of Bern, Bern, Switzerland; Department of Cardiothoracic and Vascular Surgery, Careggi University Hospital, Florence, Italy; Department of Cardiothoracic Surgery, Berufsgenossenschaftliches Universitätsklinikum Bergmannsheil, Bochum, Nordrhein-Westfalen, Germany; Cardio-Thoracic Surgery Department, Hospital de la Timone, Marseille, France; Department of Cardiovascular and Endovascular Surgery, Paracelsus Medical University, Salzburg, Austria; Department of Cardiovascular Surgery, Heart Center Hirslanden, Zurich, Switzerland; Department of Cardiothoracic Surgery, King’s College Hospital NHS Foundation Trust, London, UK; Department of Thoracic and Cardiovascular Surgery, West-German Heart and Vascular Center, University Duisburg-Essen, Essen, Germany; Department of Medicine III: Cardiology, Angiology, and Pneumology, Heidelberg University, Heidelberg, Germany; Institute for Pharmacology and Preventive Medicine, Cloppenburg, Germany; Institute for Pharmacology and Preventive Medicine, Cloppenburg, Germany; Department of Cardiac Surgery, University Hospitals Leuven, Leuven, Belgium

**Keywords:** Aortic stenosis, Surgical aortic valve replacement, Sex disparities

## Abstract

**OBJECTIVES:**

We investigated the sex-related difference in characteristics and 2-year outcomes after surgical aortic valve replacement (SAVR) by propensity-score matching (PSM).

**METHODS:**

Data from 2 prospective registries, the INSPIRIS RESILIA Durability Registry (INDURE) and IMPACT, were merged, resulting in a total of 933 patients: 735 males and 253 females undergoing first-time SAVR. The PSM was performed to assess the impact of sex on the SAVR outcomes, yielding 433 males and 243 females with comparable baseline characteristics.

**RESULTS:**

Females had a lower body mass index (median 27.1 vs 28.0 kg/m^2^; *P* = 0.008), fewer bicuspid valves (52% vs 59%; *P* = 0.036), higher EuroSCORE II (mean 2.3 vs 1.8%; *P* < 0.001) and Society of Thoracic Surgeons score (mean 1.6 vs 0.9%; *P* < 0.001), were more often in New York Heart Association functional class III/IV (47% vs 30%; *P* < 0.001) and angina Canadian Cardiovascular Society III/IV (8.2% vs 4.4%; *P* < 0.001), but had a lower rate of myocardial infarction (1.9% vs 5.2%; *P* = 0.028) compared to males. These differences vanished after PSM, except for the EuroSCORE II and Society of Thoracic Surgeons scores, which were still significantly higher in females. Furthermore, females required smaller valves (median diameter 23.0 vs 25.0 mm, *P* < 0.001). There were no differences in the length of hospital stay (median 8 days) or intensive care unit stay (median 24 vs 25 hours) between the 2 sexes. At 2 years, post-SAVR outcomes were comparable between males and females, even after PSM.

**CONCLUSIONS:**

Despite females presenting with a significantly higher surgical risk profile, 2-year outcomes following SAVR were comparable between males and females.

## Graphical abstract

**Figure:**
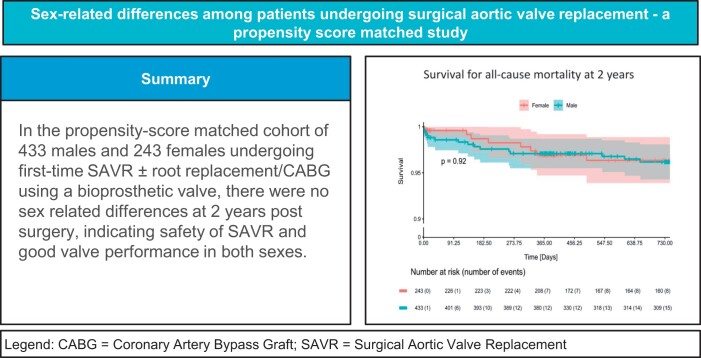

## INTRODUCTION

Surgical aortic valve replacement (SAVR) has been the gold standard treatment for aortic stenosis (AS) for decades [1]. However, a precise understanding of specific sex-related differences in baseline characteristics and post-SAVR long-term outcomes and safety remains a matter of debate [2, 3]. Although women and men share a similar prevalence of AS, SAVR is less often performed in female patients. Specific anatomical characteristics peculiar to women's hearts, such as smaller valvular size and aortic annulus/root and left ventricular outflow tract dimensions, make it technically more complicated and challenging for SAVR in women [4]. Besides, factors such as advanced age, greater frailty, smaller body size and the presence of more non-atherosclerotic comorbidities place females in a high-risk category for SAVR [3, 5, 6].

Several studies indicated that women undergoing SAVR experience worse short-term outcomes, including more in-hospital and 30-day deaths, more vascular complications and blood transfusions and increased length of hospital stays [2, 7] compared to men [2, 3, 6, 8]. Although comparable long-term survival after SAVR was observed among both sexes [8, 9], extensive research is imperative to elucidate the male-female differences in the baseline characteristics and clinical outcomes to optimize the treatment for aortic valve diseases.

## PATIENTS AND METHODS

In the present analysis, we combined data from 2 prospective, observational, multicentre registries— the INSPIRIS RESILIA Durability Registry (INDURE) and the IMPACT registry [10, 11], to study the sex-related differences in SAVR outcomes. Our goal was to report 2-year follow-up data of male and female patients undergoing SAVR using propensity score matching (PSM).

### Ethics statement

The study was approved by the institutional review board/ethics committee at each participating centre (Supplementary Material, Table S1). Written informed consent was obtained from each patient before enrolment.

### Patient population

Adult patients over 18 years of age undergoing SAVR and receiving the Edwards INSPIRIS RESILIA bioprosthesis were enrolled in the registries. In addition, patients undergoing a planned native valve replacement with or without combined aortic root replacement and/or coronary artery bypass graft (CABG) based on the preprocedural evaluation were included. Exclusion criteria included prior myocarditis within 3 months before SAVR and a double valve procedure (replacement and repair). Additionally, when a valve implant was not possible as per device instruction for use, individuals with a life expectancy <12 months and patients who were pregnant at the time of the operation were excluded.

### Objectives

The primary objective of the analysis was to compare baseline and procedural characteristics of male and female patients undergoing SAVR.

The secondary objective was to compare the sex-related differences in post-SAVR clinical outcomes as defined by the Valve Academic Research Consortium-2 [12] at the 2-year follow-up, which includes incidence of all-cause mortality, prosthetic endocarditis, thromboembolic events (stroke/transient ischaemic attack), life-threatening valve-related bleeding, repeat procedure requirement and a permanent pacemaker implant (PPI).

### Statistical analyses

Data were analysed using descriptive statistics, with categorical variables presented as absolute values and frequencies (%) and the continuous variables presented as means (standard deviation) and/or median [interquartile range (IQR)]. The percentages were calculated based on the number of patients with valid data per parameter, i.e. excluding patients with missing information. Comparisons were performed using a *t*-test or the Mann–Whitney *U* test for continuous variables, depending on distribution, and a Fisher exact or a χ^2^ test for categorical variables. Propensity scores (PS) were calculated using a generalized linear model to assess the sex-specific effects (male vs female). The following covariates were selected to calculate the PS: body mass index, valve morphology, New York Heart Association (NYHA) functional class III/IV, Canadian Cardiovascular Society (CCS) angina III/IV, diabetes mellitus, hypertension, left ventricular ejection fraction, mean transvalvular pressure gradient, previous percutaneous intervention, pacemaker, chronic obstructive pulmonary disease, dialysis, aortic valve regurgitation (moderate/severe), myocardial infarction, transient ischaemic attack/stroke, peripheral arterial disease and coronary artery disease. The 1:2 ratio matching was performed using nearest neighbour matching with a calliper width equal to 0.2 times the standard deviation of the PS logit. Post-matching, standardized mean differences were analysed for all covariates included in the PS calculation. The mean differences for all covariates post-matching were within a desirable threshold (±0.1), indicating adequate balance. Statistical analyses were performed using R version 4.3 (https://www.R-project.org/).

## RESULTS

**Figure 1:**
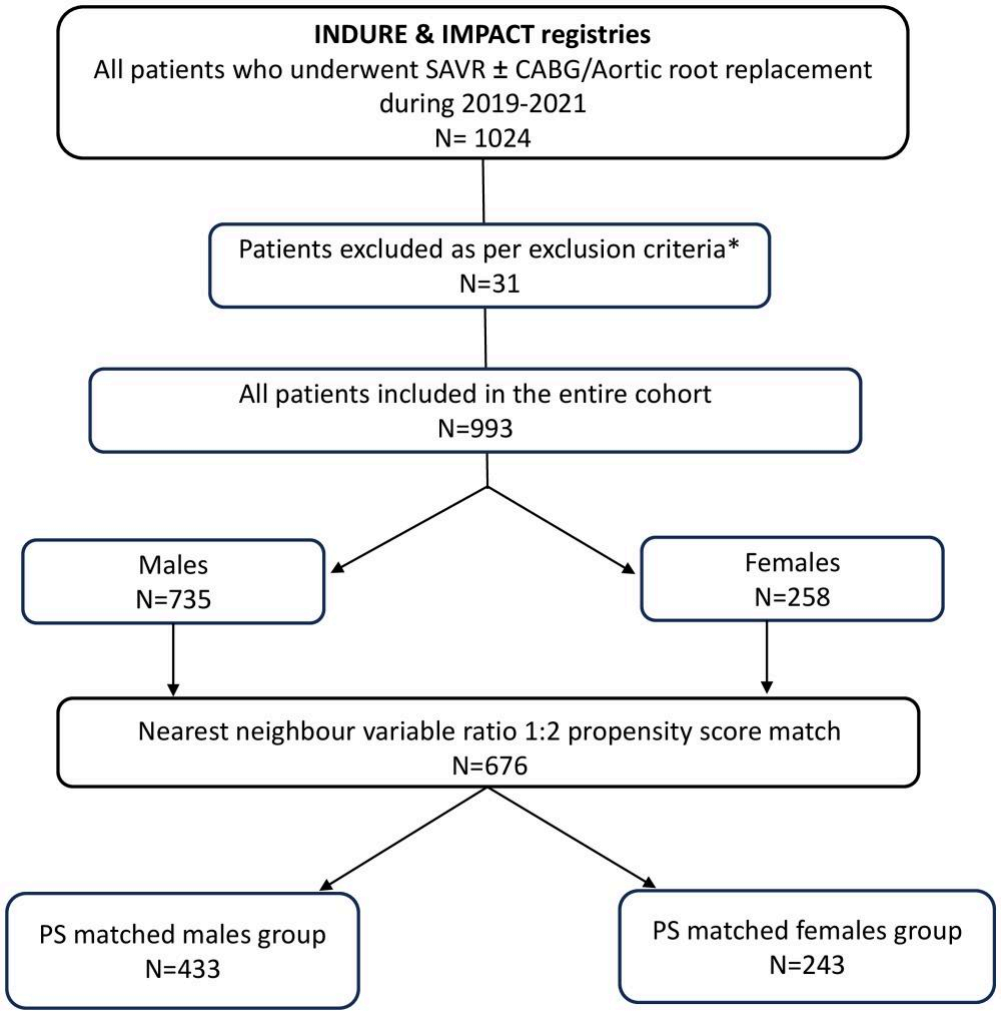
Study flow chart. CABG: coronary artery bypass graft; PS: propensity score; SAVR: surgical aortic valve replacement. *Reasons: Not meeting inclusion/exclusion criteria (*n* = 9); not receiving INSPIRIS RESILIA valve (*n* = 10); double valve procedure (replacement or repair; *n* = 10); withdrew from the study (*n* = 2).

A total of 993 patients, 735 males and 253 females, who underwent SAVR using the INSPIRIS RESILIA between 2019 and 2021 comprised the entire cohort. To assess the impact of sex on SAVR outcomes, a PSM cohort was created, resulting in a total of 676 matched pairs of 433 males and 243 females (Fig. 1).

### Patient characteristics

In the entire cohort, the female patients had a lower BMI [median 27.1 (IQR 23.4–31.0) vs 28.0 kg/m^2^ (IQR 25.2–31.0); *P* = 0.008] and were less likely to have bicuspid valves (52% vs 59%; *P* = 0.036) compared to the male patients (Table 1). Additionally, females exhibited a higher prevalence of advanced NYHA functional class III/IV symptoms (47% vs 30%; *P* < 0.001) and angina CCS class III/IV symptoms (8.2% vs 4.4%; *P* = 0.019), indicating a higher symptomatic burden at baseline. However, after PSM, the differences were not significant in any of the cases.

**Table 1:** Patient characteristics

	Full cohort					PS matched cohort				
Mean ± SD or median (IQR) or *n* (%)	Male, *N* = 735	Female, *N* = 258	SMD	95% CI	*P*-value	Male, *N* = 433	Female, *N* = 243	SMD	95% CI	*P*-value
Age, years	58.8 ± 9.2	59.8 ± 9.5	−0.11	−0.25, 0.03	0.159	59.0 ± 9.7	59.8 ± 9.5	−0.09	−0.24, 0.07	0.430
Body mass index, kg/m^2^	28.0 (25.2–31.0)	27.1 (23.4–31.0)	0.11	−0.03, 0.25	0.008	27.1 (24.7–30.2)	27.3 (23.5–31.3)	−0.05	−0.20, 0.11	0.601
Valve morphology			0.15	0.01, 0.29	0.036			0.04	−0.12, 0.19	0.647
Bicuspid	434 (59)	133 (52)				236 (55)	128 (53)			
Tricuspid	301 (41)	125 (48)				197 (45)	115 (47)			
NYHA functional class III/IV	220 (30)	121 (47)	0.36	0.22, 0.50	<0.001	169 (39)	110 (45)	0.13	−0.03, 0.28	0.114
Angina CCS III/IV	32 (4.4)	21 (8.2)	0.16	0.02, 0.30	0.019	22 (5.1)	17 (7.0)	0.08	−0.08, 0.24	0.306
EuroSCORE II, %	1.8 ± 2.0	2.3 ± 3.1	−0.18	−0.32, −0.04	<0.001	1.6 ± 1.7	2.4 ± 3.0	−0.18	−0.32, −0.04	<0.001
STS score, %	0.9 ± 2.5	1.6 ± 2.2	−0.31	−0.46, −0.17	<0.001	1.0 ± 2.3	1.7 ± 2.0	−0.33	−0.48, −0.17	<0.001
Medical history										
Diabetes mellitus	115 (16)	45 (17)	0.05	−0.09, 0.19	0.500	73 (17)	42 (17)	0.01	−0.15, 0.17	0.888
Systemic hypertension	438 (60)	148 (57)	0.05	−0.10, 0.19	0.531	243 (56)	138 (57)	0.01	−0.14, 0.17	0.866
Coronary artery disease	504 (69)	192 (75)	0.14	−0.01, 0.28	0.068	313 (72)	180 (74)	0.04	−0.12, 0.20	0.616
Myocardial infarction	38 (5.2)	5 (1.9)	0.18	0.03, 0.32	0.028	11 (2.5)	5 (2.1)	0.03	−0.12, 0.19	0.692
Peripheral vascular disease	43 (5.9)	11 (4.3)	0.07	−0.07, 0.21	0.334	21 (4.8)	11 (4.5)	0.02	−0.14, 0.17	0.849
TIA/stroke	36 (4.9)	13 (5.0)	0.01	−0.14, 0.15	0.928	19 (4.4)	11 (4.5)	0.01	−0.15, 0.16	0.933
COPD	52 (7.1)	27 (10)	0.12	−0.02, 0.26	0.083	35 (8.1)	22 (9.1)	0.03	−0.12, 0.19	0.663
PPI	13 (1.8)	4 (1.6)	0.02	−0.12, 0.16	1.000	8 (1.8)	4 (1.6)	0.02	−0.14, 0.17	1.000
Previous PCI	78 (11)	19 (7.4)	0.11	−0.03, 0.26	0.131	35 (8.1)	19 (7.8)	0.01	−0.15, 0.17	0.903
Dialysis	8 (1.1)	2 (0.8)	0.03	−0.11, 0.17	1.000	5 (1.2)	2 (0.8)	0.03	−0.12, 0.19	1.000
Echocardiography										
AV regurgitation (moderate/severe)	255 (35)	68 (27)	0.18	0.04, 0.32	0.015	128 (30)	66 (27)	0.05	−0.10, 0.21	0.508
LVEF, %	58 ± 10	60 ± 10	−0.28	−0.43, −0.14	<0.001	60 ± 9	60 ± 10	−0.04	−0.20, 0.12	0.464
Mean transvalvular pressure gradient, mmHg	43 ± 20	46 ± 21	−0.16	−0.30, −0.01	0.249	45 ± 18	46 ± 21	−0.05	−0.21, 0.12	0.690

AV: aortic valve; CCS: Canadian Cardiovascular Society; EuroSCORE: European System for Cardiac Operative Risk Evaluation; CI; confidence interval; COPD: chronic obstructive pulmonary disease; IQR: interquartile range; LVEF: left ventricular ejection fraction; NYHA: New York Heart Association; PCI: percutaneous intervention; PPI: permanent pacemaker implant; PS: propensity score; SD: standard deviation; SMD: standard mean difference; STS: Society of Thoracic Surgeons; TIA: transient ischaemic attack.

Compared to males, female patients in both cohorts exhibited significantly higher surgical risk with higher EuroSCORE II (2.3 ± 3.1% vs 1.8 ± 2.0%; *P* < 0.001) and Society of Thoracic Surgeons (STS) score (1.6 ± 2.2% vs 0.90 ± 2.5%; *P* < 0.001). Notably, these differences persisted after PSM (EuroSCORE II: 2.4 ± 3.0% vs 1.6 ± 1.7%; *P* < 0.001 and STS score: 1.7 ± 2.0% vs 1.0 ± 2.3%; *P* < 0.001). In the entire cohort, females had a lower history of myocardial infarction (1.9% vs 5.2%; *P* = 0.028) than males.

In baseline echocardiography, females exhibited a lower prevalence of moderate to severe aortic valve regurgitation (27% vs 35%; *P* = 0.015), along with a better left ventricular ejection fraction (60 ± 10% vs 58 ± 10%; *P* < 0.001) and slightly higher mean transvalvular pressure gradients (46 ± 21 vs 43 ± 20 mmHg; *P* = 0.249) compared to males. This trend did not persist after PSM.

### Procedural characteristics

In our study, both females and males had distinct AS aetiologies (*P* = 0.047), primarily showing congenital AS (51.6% in females vs 59.8% in males) followed by degenerative AS (44.6% vs 37.1%) (Supplementary Material, Table S2).

In the total cohort, minimally invasive surgery was more frequent in females (46.5% vs 38.6%; *P* = 0.027) with less concomitant CABG (10.9% vs 16.3%; *P* = 0.034) (Supplementary Material, Table S2). Notably, these differences disappeared after PSM (Table 2). Females required smaller valves [median 23.0 mm (IQR 21.0–23.0)] compared to males [median 25.0 mm (IQR 23.0–27.0)], which was significant in both total and PSM cohorts (*P* < 0.001). The majority of female patients received either 23- (44.4%) or 21- (39.9%) mm valves, whereas male patients received either 25- (37.2%) or 23- (30.7%) mm valves. There were no differences in the overall procedural time (skin-to-skin) between males and females in the matched cohort (*P* = 0.170). The first implant attempt was successful in both sexes (>99.0%), with no intraprocedural deaths.

**Table 2:** Procedural details—propensity score matched cohort

Mean ± SD or median (IQR) or *n* (%)	Male, *N* = 433	Female, *N* = 243	*P*-value
Aetiology of valve pathology			0.769
Congenital	239 (55.3)	128 (52.7)	
Degenerative	183 (42.4)	106 (43.6)	
Endocarditic	1 (0.2)	1 (0.4)	
Rheumatic	2 (0.5)	2 (0.8)	
None (no aortic stenosis)	7 (1.6)	6 (2.5)	
Isolated AVR	259 (59.8)	149 (61.3)	0.702
MIS	178 (41.1)	114 (46.9)	0.144
Concomitant procedures			
CABG	67 (15.5)	27 (11.1)	0.116
Root replacement	31 (7.2)	11 (4.5)	0.174
Supracoronary tube graft	58 (13.4)	31 (12.8)	0.814
Total operation time (skin-to-skin), min	198.3 ± 62.9 190.0 (155.0, 233.5)	191.1 ± 59.0 184.5 (148.0, 224.0)	0.170
Cross-clamp time, min	75.0 ± 26.8 70.0 (56.0, 92.0)	71.7 ± 26.3 68.0 (54.0, 88.0)	0.111
Cardiopulmonary bypass time, min	103.9 ± 39.3 98.0 (76.0, 126.0)	102.1 ± 38.1 94.0 (77.0, 121.0)	0.542
Final valve size, mm	25.0 (23.0, 25.0) 24.7 ± 2.1	23.0 (21.0, 23.0) 22.3 ± 1.5	<0.001
19	0 (0.0)	8 (3.3)	
21	32 (7.4)	97 (39.9)	
23	133 (30.7)	108 (44.4)	
25	161 (37.2)	27 (11.1)	
27	75 (17.3)	3 (1.2)	
29	32 (7.4)	0 (0.0)	
Implant details			
First implant success	432 (99.8)	242 (99.6)	1.000
Second implant with INSPIRIS RESILIA	1 (0.2)	1 (0.4)	1.000
Paravalvular leak (final)	5 (1.2)	1 (0.4)	0.427
Intraprocedural deaths	0 (0.0)	0 (0.0)	1.000

AVR: aortic valve replacement; CABG: coronary artery bypass graft; IQR: interquartile range; MIS: minimally invasive surgery; SD: standard deviation.

### Discharge characteristics

The overall hospital stay during SAVR was similar between female and male patients in the matched cohort [median 8.0 (IQR 6.0–10.0) vs 8.0 (IQR 7.0–11.5) days, *P* = 0.144; Table 3). There was no difference in the length of stay in the intensive care unit and in the duration of mechanical ventilation in both groups. A similar proportion of patients were discharged alive (females 99.6% and males 99.3%; Supplementary Material, Table S3). The majority of patients were discharged to home after the operation, followed by discharge to a rehabilitation unit or another hospital.

**Table 3:** Discharge details—propensity matched cohort

Mean ± SD or median (IQR) or *n* (%)	Male, *N* = 433	Female, *N* = 243	*P*-value
Hospital stay, days	9.0 ± 4.5 8.0 (6.0, 10.0)	9.9 ± 6.5 8.0 (7.0, 11.5)	0.144
Discharged alive	428 (99.3)	242 (99.6)	1.000
Discharge to			0.428
Home	257 (59.6)	151 (62.1)	
Other hospital	33 (7.7)	25 (10.3)	
Rehabilitation unit	135 (31.3)	66 (27.2)	
Other	3 (0.7)	0 (0.0)	
Death	3 (0.7)	1 (0.4)	
ICU stay, h	46.4 ± 54.7 24.0 (21.0, 48.0)	52.0 ± 59.0 25.0 (22.0, 62.0)	0.449
Mechanical ventilation, h	11.9 ± 39.5 7.0 (4.0, 10.0)	10.1 ± 15.0 7.0 (5.0, 10.0)	0.609

ICU: intensive care unit; IQR: interquartile range; SD: standard deviation.

### Clinical outcomes

**Figure 2:**
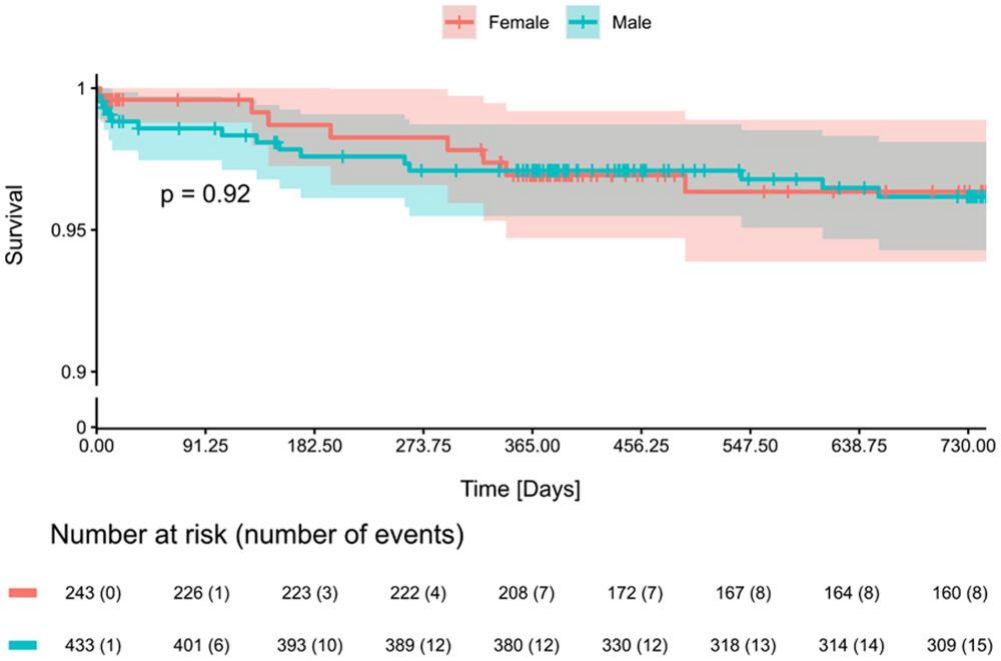
Kaplan–Meier survival curve at 2-year all-cause mortality stratified by sex—propensity score matched cohort. PS: propensity score.

Both in the entire and the PS-matched cohorts, no significant differences were observed in the incidence of clinical outcomes at 2 years, including endocarditis, thromboembolic events, valve-related dysfunction, repeated procedure, permanent pacemaker implant and valve-related bleeding between males and females undergoing SAVR ± CABG/root replacement (Supplementary Material, Table S4; Table 4) as well as in patients undergoing isolated AVR (Supplementary Material, Table S5). The 2-year survival rate in the PS-matched cohort was 96.2% [95% confidence interval (CI): 94.3–98.1%] in males and 96.3% (CI: 93.9–98.9%) in females (*P* = 0.920); no differences were observed in the total cohort (Fig. 2, Supplementary Material, Fig. S1). Although the rate of valve thrombosis at 2 years seemed to be higher in females (1.3% vs 0.4% in the PS-matched cohort), the difference did not reach statistical significance (*P* = 0.093).

**Table 4:** Two-year clinical outcomes—propensity score matched cohort

	Early (≤30 days)		Late (>30 days to 2 years)		Freedom from events at 2 years, % (95% CI)		
*n* (%)	Male, *N* = 433	Female, *N* = 243	Male, 732 vy	Female, 400 vy	Male	Female	*P*-value
All-cause mortality	5 (1.2)	1 (0.4)	10 (1.4)	7 (1.8)	96.2 (94.3, 98.1)	96.3 (93.9, 98.9)	0.920
Cardiovascular-related	5 (1.2)	1 (0.4)	7 (1.0)	3 (0.8)	97.0 (95.4, 98.7)	98.1 (96.3, 100.0)	0.365
Valve-related	2 (0.5)	0 (0)	5 (0.7)	2 (0.5)	98.3 (97.0, 99.6)	98.9 (97.5, 100.0)	0.394
Valve-related—unknown	1 (0.2)	0 (0)	2 (0.3)	4 (1.0)	99.1 (98.2, 100.0)	98.1 (96.2, 100.0)	0.233
Prosthesis endocarditis	0 (0)	0 (0)	4 (0.5)	2 (0.5)	99.0 (98.0, 100.0)	99.0 (97.5, 100.0)	0.909
Thromboembolic events	11 (2.5)	4 (1.6)	4 (0.5)	4 (1.0)	95.9 (93.8, 97.9)	95.8 (93.0, 98.7)	0.967
Stroke	7 (1.6)	4 (1.6)	0 (0)	1 (0.3)	98.1 (96.7, 99.5)	97.4 (95.2, 99.7)	0.594
Valve thrombosis	0 (0)	0 (0)	3 (0.4)	5 (1.3)	99.7 (99.1, 100.0)	98.0 (96.0, 100.0)	0.093
Valve-related dysfunction	1 (0.2)	0 (0)	3 (0.4)	5 (1.3)	99.5 (98.8, 100.0)	98.6 (97.1, 100.0)	0.196
Repeat procedure	1 (0.2)	0 (0)	0 (0)	3 (0.8)	99.8 (99.3, 100.0)	99.0 (97.5, 100.0)	0.096
Permanent pacemaker	18 (4.2)	9 (3.7)	2 (0.3)	2 (0.5)	95.2 (93.2, 97.3)	95.4 (92.7, 98.1)	0.944
Valve-related bleeding	43 (9.9)	29 (11.9)	2 (0.3)	3 (0.8)	89.5 (86.7, 92.5)	86.6 (82.4, 91.1)	0.282

CI: confidence interval; vy: valve years.

The majority of patients requiring a repeat procedure at the 2-year follow-up in our study did so due to the presence of the endocarditis; in 1 patient, a repeat procedure was due to valve thrombosis whereas another one had a moderate paravalvular leak. One patient underwent a valve-in-valve procedure due to AS. Furthermore, all patients reporting prosthetic valve thrombosis at 2 years in our study either initiated or changed anticoagulation therapy and had a regression and good prosthesis function as shown by the decreased mean pressure gradient in the follow-up echocardiogram. For 1 patient, valve thrombosis was reverted despite the absence of anticoagulant therapy. Therefore, the presence of the valve thrombosis was mostly subclinical and did not lead to detrimental clinical consequences after SAVR using a biosprosthetic valve.

## DISCUSSION

Key findings of this propensity score matched study based on 2-year data from the INDURE and IMPACT registries were (i) females exhibited higher surgical risk (EuroSCORE II and STS scores), had higher symptomatic burden (NYHA functional class III/IV and angina CCS III/IV) than males with similar comorbidity prevalences; (ii) females received smaller valves than males, with a median diameter of 23 mm compared to 25 mm in males; (iii) both male and female patients experienced similar hospital lengths of stay in the intensive care unit after SAVR; (iv) patients demonstrated comparable outcomes at 2 years after SAVR, suggesting that sex-related differences observed at baseline did not impact clinical outcomes.

In the overall population (*n* = 993), the proportion of female patients undergoing SAVR from 2019 to 2021 was lower compared to the proportion of male patients [258 (26.0%) vs 735 (74.0%)]. This disparity suggests a lower incidence of SAVR in females than males, consistent with findings reported in prior literature [2, 3, 7]. Despite a similar prevalence of AS [13], the specific factors contributing to the lower rate of SAVR in women remain unclear. Several studies have proposed possible explanations, such as the insidious onset of the disease in females, delayed diagnosis, conservative management, less frequent referrals to specialists and fewer diagnostic tests conducted among women [2, 14, 15]. However, it is important to note that our study did not focus on the male–female disparity in the incidence of SAVR, the time that elapsed between diagnosis and intervention or the urgency of SAVR, which represents a limitation of our findings.

Several previously published studies [2, 9, 16–18] have investigated sex-related differences in patients undergoing SAVR. These studies consistently reported that females undergoing SAVR tended to be older, exhibited advanced NYHA symptoms and angina symptoms and had higher surgical risks compared to males. Our study results align with these findings, because females exhibited significantly higher EuroSCORE II and STS scores in both cohorts (*P* < 0.001), indicating a greater surgical risk profile in females. Nevertheless, there was no significant difference in age between males and females in our study, and they were younger (both sexes) than the populations studied earlier [15, 17, 18]. Furthermore, in our cohort, females showed advanced NYHA functional class III/IV and angina CCS III/IV symptoms compared with the males (*P* < 0.001), indicating a heightened cardiac risk and symptomatic burden than male patients; this trend was consistent with the observations of previous studies [9, 17, 18]. Contrary to the lower comorbidity prevalence observed among female patients undergoing SAVR in the PARTNER trial [15] and the study by Triboulloy *et al.* [17], our study did not reveal significant differences between males and females. Nonetheless, our study did note a higher prevalence of previous MIs among males, aligning with the findings of Hernandez-Vaquero *et al.* [16] and Tribouilloy *et al.* [17].

Notably, a significant difference was observed in implanted valve sizes between the sexes, with females being implanted with smaller valves than males (median diameter 23 vs 25 mm; *P* < 0.001). This difference is attributed to anatomical differences, with women typically having smaller hearts and aortic annuli [19] than men. Consequently, the need for smaller aortic bioprostheses in women has been recognized in previous research and is associated with increased risk in SAVR [20]. Therefore, it underscores the importance of selecting valve size based on precise in vivo measurements of the patient's specific annular dimensions.

Despite significant differences in baseline characteristics, indicating a high surgical risk among females in our study, the 2-year outcomes after SAVR revealed comparable outcomes in both sexes. However, the existing literature shows varied findings. For instance, a study by Kulik *et al.* comparing long-term outcomes of SAVR over 5.6 years reported a significantly lower reoperation rate in women (comorbidity-adjusted hazard ratio 0.4; 95% CI: 0.2–0.9) and a higher incidence of late stroke (hazard ratio 1.7; 95% CI: 1.1–2.7) compared to men, indicating that sex-related differences in long-term SAVR outcomes exist [21]. Despite these discrepancies, women exhibited better overall long-term survival than men in their study. Similarly, findings from the Simvastatin and Ezetimibe in Aortic Stenosis (SEAS) study, with a median follow-up of 4 years, revealed that females exhibited lower total mortality and a reduced rate of ischaemic cardiovascular events compared to men, independent of confounding factors, despite similar AS progression and greater severity in females based on echocardiographic indices [22]. On the other hand, another baseline-matched retrospective study reported comparable long-term survival benefits in females at a 5-year follow-up. However, men faced a higher risk of bleeding, endocarditis and early reoperation after SAVR [9]. Thus, collectively, these studies suggest that female sex does not significantly impact the long-term survival of SAVR when preoperative characteristics are adjusted between the 2 sexes.

### Limitations

Our study did not capture data on matching-based postoperative ventricular remodelling and prosthetic valve performance following surgery, which could elucidate casual factors impacting the outcome for males and females. Additionally, we did not gather information on the timing of intervention and the urgency of SAVR. Furthermore, our study lacks data on prosthetic–patient mismatch, a common complication of cardiac surgery [23].

## CONCLUSION

Women undergo SAVR less frequently and exhibit a higher risk profile, posing unique challenges for cardiac surgeons. Nevertheless, our analysis reveals that the 2-year clinical outcomes of SAVR are similar between the sexes when baseline characteristics are matched. These findings highlight the importance of considering sex-related factors in evaluating surgical risk and treatment strategies for patients having SAVR.

## Supplementary Material

ivae140_Supplementary_Data

## Data Availability

The data sets generated and analysed during the current study may be available from the corresponding author upon reasonable request.

## SUPPLEMENTARY MATERIAL

**Conflicts of interest:** Andreas Zierer, Ruggero De Paulis, Farhad Bakhtiary, Thierry Bourguignon, Nikolaos Bonaros, Michael Borger, Justus Strauch, Peter Bramlage and Bart Meuris have received lecture fees and/or research support from Edwards Lifesciences. Richard Feyrer, Andreas Liebold and Andreas Vötsch have received lecture fees/proctoring fees from Edwards Lifesciences. Beate Botta received research support from Edwards Lifesciences for their institution. The institutions of all authors representing study centres have received patient inclusion-based funding; these authors have no conflicts of interest to disclose.

## Notes

Presented at the EACTS 2023, Vienna, 07 October 2023.

## ABBREVIATIONS

AS: Aortic stenosis
CABG: Coronary artery bypass graft
CCS: Canadian Cardiovascular Society
IQR: Interquartile range
NYHA: New York Heart Association
PSM: Propensity score matching
SAVR: Surgical aortic valve replacement
STS: Society of Thoracic Surgeons
