# Supplementary material for: Sex-related differences among patients undergoing surgical aortic valve replacement—a propensity score matched study
Source: Interdiscip Cardiovasc Thorac Surg. 2024 Aug 10;39(2):ivae140. doi: 10.1093/icvts/ivae140 (PMC11341123; doi:10.1093/icvts/ivae140)
Supplement: ivae140_Supplementary_Data [file ivae140_supplementary_data.docx]

**SUPPLEMENT**

**Figure S1:** Kaplan-Meier survival curve at 2-year all-cause mortality stratified by sex – Total cohort

| 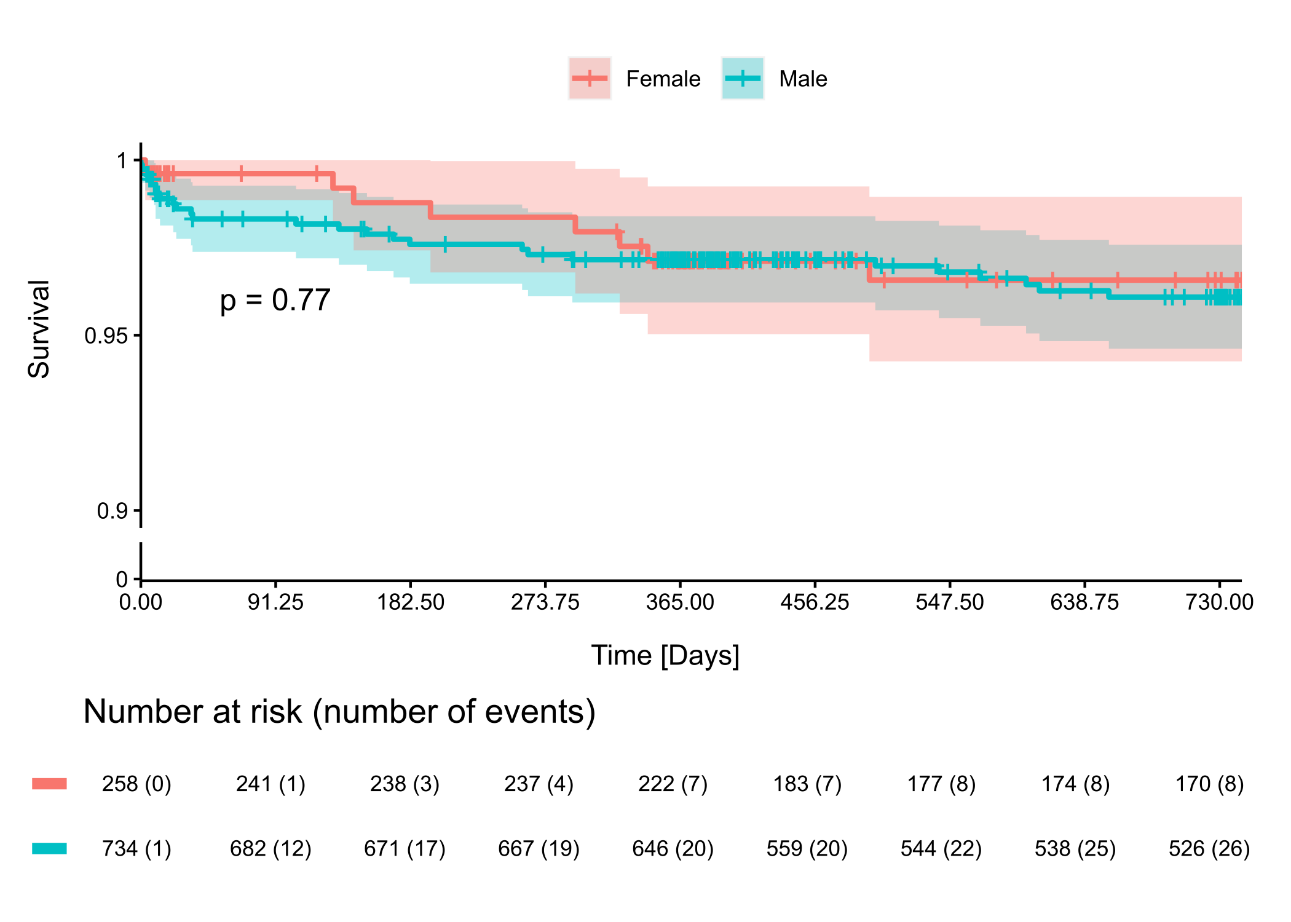 |
| --- |

**Table S1:** Details of Ethical Approvals from Centres

| Name of institutional review board/Ethics committe | Ethical approval number |
| --- | --- |
| Comité de protection des personne ile-de-france vii | 2019-a00794-53 |
| Ethik-commission medizinische universität wein | 1117/2019 |
| Comitato etico regionale per la sperimentazione  Clinica della regione toscana | 15402_oss |
| Assistance publique hopitaux de Marseille | 2206001 v 0 |
| Ethik-kommission albert ludwigs universität freiburg | 104/19 |
| Ethik-kommission universität leipzig, medizinische fakultät, leipzig | 028/19-ek |
| Ethische commissie onderzoek uz/ku leuven, belgium | B322201940075 |
| Comitato etico degli irccs istituto europeo di oncologia e centro cardiologico monzino | R1059/19-ccm 1123 |
| Institut de cardiologie de montréal | Mp-33-2020-2648 |
| Que el ceim hospital virgen de la arrixaca | Htv-r18-ind |
| Institut universitaire de cardiologie et de pneumologie de québec-u Niversité laval | Meo-33-2020-3291, mp-33-2020-2648, 21886 |
| Comitato etico lazio 2 | Studio 46.19 |
| Medische ethische toetsings cpmmisie erasmus mc | Wt/aj/mec-2019-0082 |
| Servizio coordinamento comitato etico campania sud via marconi, 66 80049 torre del greco-napoli | Prot./scce n.83233 |
| Universitätsklinikum st.pölten | Gs4-ek-3/156-2019 |
| Ethik-kommission derstadt wein | Hvt-r18-331 |
| Kings college hospital london | 19/lo/0439 |
| Ethik-kommission des landes oberösterreich | 1190/2019 |
| Ärztekammer niedersachsen | Grae/205/2020 |
| Ethik-kommission der med. Fakultät der rub gesundheitscampus 33, 44801 bochum | 19-6804 mpg |
| Ethik-kommission med. Fakultät der heinrich-heine universität düsseldorf | 2020055564 |
| Een santeon zeikenhuis | Nwmo-2020.204 |
| Ethik-kommission med. Fakultät der universität duisburg essen | 20-9145-bo |
| Ethik-kommission fachbereich medizin frankfurt goethe- universität | 20-972 |
| Ethik-kommission der medizinischen universität innsbruck | 1009/2020 |
| Ethik-kommission landesärztekammer rheinland-pfalz | 2020-14823-mpg |
| Ethik-kommission medizinische fakultät universität leipzig | 2019350 |
| Ethik-kommission des landes oberösterreich | 1190/2019 |
| Ethik-kommission der bayerischen landesärztekammer | Mb bo 19044 |
| Medizinische ethik-kommission carl von ossietzky universität oldenburg | 2020-019 |
| Ethik-kommission ärztekammer nordrhein | 2029350 |
| Ethik-kommission ulm universität | 436/19 |
| Ethik-kommission der universität witten/herdecke | 91/2022 |
| Ethik-kommission der universität würzburg | 2/20_mpz-sc |
| Kantonale ethik-kommission zürich | 2019-02420 |

**Table S2:** Procedure characteristics – Total cohort

| Mean±SD or median (IQR) or n (%) | Male, N=735 | Female, N=258 | p-value |
| --- | --- | --- | --- |
| Etiology of valve pathology |  |  | 0.047 |
| Congenital | 439 (59.8) | 133 (51.6) |  |
| Degenerative | 272 (37.1) | 115 (44.6) |  |
| Endocarditic | 1 (0.1) | 1 (0.4) |  |
| Rheumatic | 2 (0.3) | 3 (1.2) |  |
| None (no aortic stenosis) | 20 (2.7) | 6 (2.3) |  |
| Unknown | 1 | 0 |  |
| Isolated AVR | 419 (57.0) | 160 (62.0) | 0.160 |
| MIS | 284 (38.6) | 120 (46.5) | 0.027 |
| Concomitant procedures |  |  |  |
| CABG | 120 (16.3) | 28 (10.9) | 0.034 |
| Root replacement | 54 (7.3) | 12 (4.7) | 0.135 |
| Supracoronary tube graft | 109 (14.8) | 33 (12.8) | 0.421 |
| Duration of intervention |  |  |  |
| Total operation time (skin-to-skin), min | 202.0±69.8  193.0 (157.0, 238.0) | 190.3±58.7  183.0 (147.3, 221.5) | 0.022 |
| Cross-clamp time, min | 76.4±27.8  71.5 (56.0, 93.0) | 71.4±26.0  67.0 (53.8, 87.3) | 0.009 |
| Cardiopulmonary bypass time, min | 105.9±41.0 98.00 (77.0, 129.0) | 101.7±38.6 93.00 (76.0, 121.0) | 0.145 |
| Final valve size, mm | 25.0 (23.0, 27.0)  25.0±2.0 | 23.0 (21.0, 23.0)  22.3±1.5 | <0.001 |
| 19 mm | 0 (0.0) | 9 (3.5) |  |
| 21 mm | 40 (5.4) | 102 (39.5) |  |
| 23 mm | 211 (28.7) | 116 (45.0) |  |
| 25 mm | 274 (37.3) | 28 (10.9) |  |
| 27 mm | 151 (20.5) | 3 (1.2) |  |
| 29 mm | 59 (8.0) | 0 (0.0) |  |
| Implantation details |  |  |  |
| 1^st^ Implantation success | 732 (99.6) | 257 (99.6) | >0.999 |
| 2^nd^ implantation with INSPIRIS Resilia | 3 (0.4) | 1 (0.4) | >0.999 |
| Paravalvular leak (final) | 8 (1.1) | 1 (0.4) | 0.460 |
| Intraprocedural mortality | 0 (0.0) | 0 (0.0) | >0.999 |

Legend: CABG; coronary artery bypass graft; IQR, interquartile range; MIS, minimally invasive surgery; PS, propensity score; SD, standard deviation

**Table S3:** Discharge details – Total cohort

| Mean±SD or Median (IQR) or n (%) | Male, N=735 | Female, N=258 | p-value |
| --- | --- | --- | --- |
| Hospital stay, days | 9.3±5.29  8.0 (7.0, 11.0) | 9.9±6.9 8.0 (6.0, 11.0) | 0.526 |
| Discharged alive | 725 (99.0) | 257 (99.6) | 0.688 |
| Discharge to |  |  | 0.618 |
| Death | 7 (1.0) | 1 (0.4) |  |
| Home | 442 (60.4) | 161 (62.4) |  |
| Other hospital | 60 (8.2) | 26 (10.1) |  |
| Rehabilitation unit | 220 (30.1) | 70 (27.1) |  |
| Other | 3 (0.4) | 0 (0.0) |  |
| Stay in ICU, hours | 49.6±62.2  25.0 (21.0, 49.0) | 50.6±57.7  24.0 (21.0, 60.0) | 0.911 |
| Mechanical ventilation, hours | 15.0±54.9  7.00 (5.0, 10.0) | 10.0±14.6  7.0 (5.0, 10.0) | 0.607 |

Legends: ICU; intensive care unit; IQR, interquartile range; LoS, length of stay; PS, propensity score; SD, standard deviation

**Table S4:** Two-year clinical outcomes – Total cohort

|  | **Early (≤30 days)** | | **Late (>30 days to 2 year)** | | **Freedom from events at 2 years**  **% (95%CI)** | |  |
| --- | --- | --- | --- | --- | --- | --- | --- |
| n (%) | Male,  N=735 | Female,  N=258 | Male,  1249 vy | Female,  425 vy | Male | Female | p-value |
| All-cause mortality | 10 (1.4) | 1 (0.4) | 16 (1.3) | 7 (1.6) | 96.1 (94.6, 97.6) | 96.6 (94.3, 98.9) | 0.765 |
| Cardiovascular-related | 10 (1.4) | 1 (0.4) | 10 (0.8) | 3 (0.7) | 97.1 (95.8, 98.4) | 98.2 (96.5, 100.0) | 0.302 |
| Valve-related | 4 (0.5) | 0 (0) | 7 (0.6) | 2 (0.5) | 98.4 (97.4, 99.3) | 99.0 (97.6, 100.0) | 0.389 |
| Valve-related - Unknown | 3 (0.4) | 0 (0) | 3 (0.2) | 4 (0.9) | 99.0 (98.3, 99.8) | 98.2 (96.4, 100.0) | 0.304 |
| Prosthesis endocarditis | 1 (0.1) | 0 (0) | 15 (1.2) | 2 (0.5) | 97.4 (96.2, 98.7) | 99.0 (97.7, 100.0) | 0.161 |
| Thromboembolic events | 25 (3.4) | 4 (1.6) | 9 (0.7) | 4 (0.9) | 94.4 (92.6, 96.3) | 96.0 (93.4, 98.8) | 0.385 |
| Stroke | 15 (2.0) | 4 (1.6) | 0 (0) | 1 (0.2) | 97.6 (96.4, 98.8) | 97.6 (95.5, 99.7) | 0.980 |
| Valve thrombosis | 0 (0) | 0 (0) | 5 (0.4) | 5 (1.2) | 99.5 (99.0, 100.0) | 98.1 (96.2, 100.0) | 0.068 |
| Valve-related dysfunction | 2 (0.3) | 0 (0) | 7 (0.6) | 5 (1.2) | 98.9 (98.2, 99.7) | 98.7 (97.3, 100.0) | 0.376 |
| Repeated procedure | 3 (0.4) | 0 (0) | 5 (0.4) | 3 (0.7) | 98.8 (97.9, 99.6) | 99.0 (97.7, 100.0) | 0.898 |
| Permanent pacemaker | 31 (4.2) | 9 (3.5) | 8 (0.6) | 2 (0.5) | 94.5 (92.8, 96.2) | 95.7 (93.2, 98.2) | 0.514 |
| Valve-related bleeding | 79 (10.7) | 30 (11.6) | 3 (0.2) | 3 (0.7) | 88.7 (86.5, 91.1) | 87.0 (83.0, 91.3) | 0.504 |

Legends: CI, confidence interval; vy, valve years

**Table S5:** 2-year clinical outcome in patient isolated aortic valve replacements (Male vs. female)

|  | **Freedom from events at 2 years**  **% (95%CI)** | |  |
| --- | --- | --- | --- |
| n (%) | Male  (n=259) | Female  (n=149) | p-value |
| All-cause mortality | 97.0 (94.7, 99.2) | 98.4 (96.1, 100.0) | 0.367 |
| Cardiovascular-related | 97.5 (95.5, 99.5) | 99.1 (97.3, 100.0) | 0.217 |
| Valve-related | 98.0 (96.2, 99.7) | 99.1 (97.3, 100.0) | 0.304 |
| Prosthesis endocarditis | 99.2 (98.0, 100.0) | 99.1 (97.3, 100.0) | 0.900 |
| Thromboembolic events | 96.3 (93.8, 98.8) | 98.3 (95.9, 100.0) | 0.323 |
| Stroke | 97.2 (95.0, 99.4) | 98.3 (95.9, 100.0) | 0.560 |
| Valve thrombosis | 99.5 (98.6, 100.0) | 97.7 (95.1, 100.0) | 0.461 |
| Valve-related dysfunction | 99.5 (98.7, 100.0) | 98.6 (96.6, 100.0) | 0.857 |
| Repeated procedure | 99.6 (98.8, 100.0) | 99.1 (97.3, 100.0) | 0.694 |
| Permanent pacemaker | 97.3 (95.3, 99.3) | 96.6 (93.7, 99.6) | 0.736 |
| Valve-related bleeding | 91.4 (88.0, 94.9) | 89.2 (84.4, 94.4) | 0.449 |

Legend: C confidence interval;
